# Analogs of the Heat Shock Protein 70 Inhibitor MKT-077 Suppress Medullary Thyroid Carcinoma Cells

**DOI:** 10.3390/ijms23031063

**Published:** 2022-01-19

**Authors:** Seung-Keun Hong, Dmytro Starenki, Oleta T. Johnson, Jason E. Gestwicki, Jong-In Park

**Affiliations:** 1 Department of Biochemistry, Medical College of Wisconsin, Milwaukee, WI 53226, USA; skhong@mcw.edu (S.-K.H.); dstarenki@hudsonalpha.org (D.S.); 2 Department of Pharmaceutical Chemistry, University of California San Francisco, San Francisco, CA 94158, USA; oleta.johnson@ucsf.edu (O.T.J.); jason.gestwicki@ucsf.edu (J.E.G.)

**Keywords:** medullary thyroid carcinoma, mitochondria, mortalin, HSPA9, MKT-077, JG-98

## Abstract

Medullary thyroid carcinoma (MTC) is a neuroendocrine tumor mainly caused by mutations in the *RET* proto-oncogene. We previously demonstrated that depletion of the mitochondrial molecular chaperone, mortalin, can effectively suppress human MTC cells in culture and in mouse xenografts, by disrupting mitochondrial bioenergetics and subsequently inducing apoptosis and RET downregulation. Similar effects were induced by MKT-077, a water-soluble rhodocyanine dye analog known to inhibit mortalin, but with notable toxicity in animals. These observations led us to evaluate recently developed MKT-077 analogs that exhibited higher selectivity to HSP70 proteins and improved bioavailability. We validated the MTC cell-suppressive effects of mortalin depletion in three-dimensional cultures of the human MTC lines, TT, and MZ-CRC-1, and then evaluated different MKT-077 analogs in two- and three-dimensional cell cultures, to show that the MKT-077 analogs, JG-98 and JG-194, effectively and consistently inhibited propagation of TT and MZ-CRC-1 cells in these cultures. Of note, these compounds also effectively suppressed the viability of TT and MZ-CRC-1 progenies resistant to vandetanib and cabozantinib. Moreover, JG-231, an analog with improved microsomal stability, consistently suppressed TT and MZ-CRC-1 xenografts in mice. These data suggest that mortalin inhibition may have therapeutic potential for MTC.

## 1. Introduction

Medullary thyroid carcinoma (MTC) is a relatively rare endocrine tumor that originates from parafollicular C-cells of the thyroid gland, accounting for about 5% of all thyroid cancers [1]. MTC occurs sporadically or in a hereditary form, i.e., familial MTC and multiple endocrine neoplasia type 2 syndrome, and progresses slowly. Surgical resection is the only curative therapy for MTC, but this is not effective for metastatic or recurring MTC. Therefore, a molecularly targeted therapy is necessary to treat patients with inoperable progressive MTC. The etiology of MTC is mainly attributed to mutations in the receptor tyrosine kinase, rearranged during transfection (*RET*), although other oncogenic mutations are also detected in MTC [2,3,4]. Approximately 95% of hereditary MTC and 50% of sporadic MTC cases present a mutation in the extracellular cysteine-rich receptor domain or the intracellular tyrosine kinase domain of RET (reviewed in [5]). As such, vandetanib (brand name, Caprelsa) and cabozantinib (brand name, Cabometyx), which inhibit RET and other tyrosine kinase receptors, and more advanced RET inhibitors, praseltinib (brand name, Gavreto) and selpercatinib (brand name, Retevmo), are now available to treat patients with advanced MTC [6,7,8,9]. Nevertheless, not all patients are responsive to these drugs, and additional therapeutic modalities are necessary.

Mitochondrial metabolism is often reprogrammed in cancer in response to the increased demands of tumor cells for energy and building blocks [10]. This provides a rationale to target mitochondria for cancer therapy [11,12], which may also be applicable for designing a therapeutic strategy for MTC. For example, we previously showed that mitochondria-targeted metabolic interfering agents such as triphenyl-phosphonium-carboxy-proxyl and mitoquinone (trade name MitoQ) can effectively suppress human MTC cells, including their vandetanib- and cabozantinib-resistant progenies [13,14]. We also identified mortalin (GRP75/HSPA9), a mitochondrial chaperone of HSP70 family, as a specific target to trigger mitochondria-originated death in MTC cells [15]. Briefly, we showed mortalin upregulation in human MTC specimens and that mortalin depletion can induce MTC cell death accompanied by disrupted mitochondrial bioenergetics, redox imbalance, and RET downregulation [15]. Consistent with this, similar growth inhibitory effects were induced in MTC cells by MKT-077 (1-ethyl-2-[[3-ethyl-5-(3-methylbenzothiazolin-2-yliden)]-4-oxothiazolidin-2-ylidenemethyl] pyridinium chloride), a water-soluble lipophilic cationic rhodocyanine dye, known for its tendency to partition into mitochondria and ability to inhibit a few HSP70 family members, including mortalin and HSC70 [16,17]. These findings suggest that mortalin inhibition is a potential strategy to trigger mitochondrial death in MTC cells. Nevertheless, MKT-077 did not pass human clinical trials, due to renal toxicity [18], and more advanced inhibitors are required to target mortalin for clinical purposes.

Recently, different series of MKT-077 derivatives with improved potency and metabolic stability have been developed [19,20]. However, these analogs have not yet been tested for activity in MTC models. In this study, we evaluated these MKT-077 analogs for their potency to suppress the viability of TT and MZ-CRC-1 cells, which are the only available human cell line models for MTC. TT and MZ-CRC-1 harbor *RET*^C634W^ extracellular domain mutation and *RET*^M918T^ kinase domain mutation, respectively. Our data demonstrate that certain benzothiazole rhodacyanine derivatives of MKT-077 can effectively suppress these MTC cells in vitro and in vivo.

## 2. Results

### 2.1. Benzothiazole Rhodacyanine Derivatives of MKT-077 Effectively Suppress the Viability of Human MTC Cell Lines, TT and MZ-CRC-1, in Cultures

**Figure 1:**
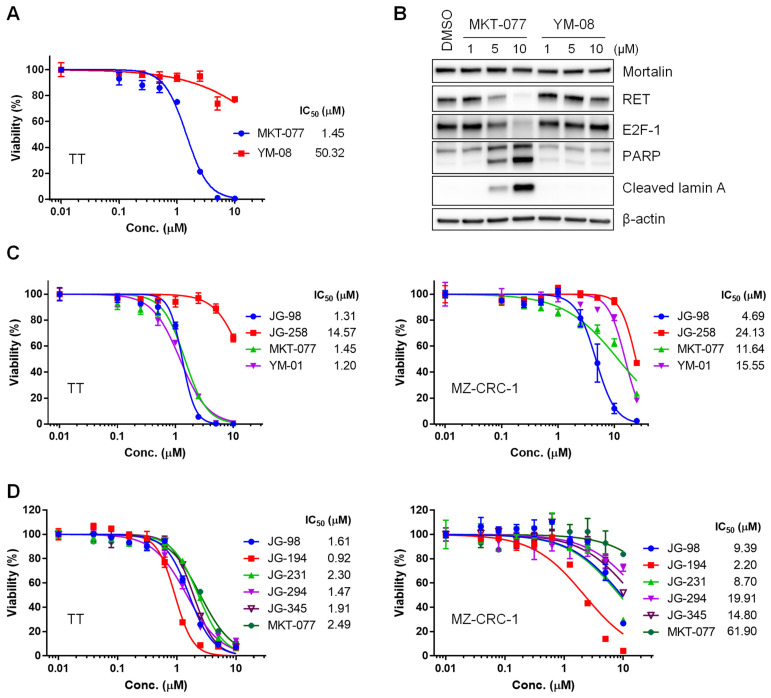
Effects of MKT-077 derivatives in TT and MZ-CRC-1 cells. (**A**) TT cells in 24-well plates were treated with serially increasing doses of MKT-077 and YM-08 for 48 h. Cells were then allowed to recover in drug-free fresh medium for 48 h, prior to determining cell viability by MTT assay, as described in the Materials and Methods. Data (mean ± SEM, *n* = 3) are expressed as the percentage of vehicle-treated control. The IC_50_ values were calculated by PRISM. (**B**) Western blot analysis of total lysates of TT cells treated with MKT-077 and YM-08 for 48 h. β-actin is the control for equal protein loading. Equal volume of DMSO was used as the vehicle control. Blots are representative of two independent experiments. (**C**) IC_50_ analysis in TT and MZ-CRC-1 cell cultures treated with indicated inhibitors for 72 h. Cell viability was determined by MTT assay. Data (mean ± SEM, *n* = 3) are expressed as the percentage of vehicle-treated control. (**D**) IC_50_ analysis in TT and MZ-CRC-1 cell cultures treated with indicated inhibitors for 72 h. Cell viability was determined by MTT assay. Data (mean ± SEM, *n* = 3) are expressed as the percentage of vehicle-treated control. 95% Confidence Intervals in TT were 1.474 to 1.772 (JG-98), 0.848 to 1.019 (JG-194), 2.095 to 2.541 (JG-231), 1.315 to 1.653 (JG-294), 1.785 to 2.052 (JG-345), and 2.261 to 2.758 (MKT-077). While, 95% Confidence Intervals in MZ-CRC-1 were 6.070 to 14.54 (JG-98), 1.592 to 3.047 (JG-194), 5.917 to 12.80 (JG-231), 14.11 to 28.09 (JG-294), 10.92 to 20.06 (JG-345), and 29.85 to 128.4 (MKT-077).

Due to its lipophilic cationic nature, MKT-077 is enriched in mitochondria. We determined whether this characteristic is necessary for MKT-077 to suppress MTC cell viability using YM-08, a neutral analog of MKT-077, in which the cationic pyridinium is replaced with a neutral pyridine [19]. Accordingly, YM-08 cannot partition into mitochondria, although it retains the ability to interact with HSP70 proteins, even with higher affinity in vitro [19]. The chemical structures of these compounds are depicted in Figure S1. When tested using equimolar doses, YM-08 did not suppress TT cell viability as effectively as MKT-077 (Figure 1A). Of note, the IC_50_ values of MKT-077 and YM-08 were more than 40-fold different in TT cells. YM-08 also did not induce downregulation of RET and the S-phase transcription factor E2F-1, or trigger the cleavage of poly (ADP-ribose) polymerase (PARP) and lamin A (Figure 1B). These data suggest that mitochondrial partitioning of MKT-077 is especially critical for its anti-proliferative effects in TT cells, which is consistent with our previous observation that mortalin is predominantly localized in mitochondria in TT cells [15].

MKT-077 was recently subjected to a medicinal chemistry campaign, to identify its derivatives with improved binding to HSP70 paralogs. This effort led to the identification of JG-98 [21]. We evaluated JG-98 for its potency to suppress the viability of TT and MZ-CRC-1 cells in comparison with MKT-077, YM-01 (a close derivative of MKT-077 [19]), and JG-258 (an inactive control compound, wherein the benzothiazole was truncated [20]). Chemical structures of these compounds are depicted in Figure S1. Our analyses of the IC_50_ values of these compounds revealed that JG-98 and YM-01 can suppress the viability of TT cells as effectively as MKT-077 (Figure 1C, left). Importantly, the inactive control, JG-258, was inactive. We previously showed that MZ-CRC-1 cells are not as sensitive as TT cells to MKT-077 [22]. Consistent with this, the IC_50_ value of MKT-077 was only about 2-fold lower than that of the control compound, JG-258, in MZ-CRC-1 cells (Figure 1C, right). YM-01, whose structure is very close to MKT-077 (Figure S1), also did not exhibit a higher efficacy than MKT-077 (Figure 1C, right). However, in marked contrast, JG-98 suppressed the viability of MZ-CRC-1 cells (Figure 1C, right), with an IC_50_ value less than 5 µM.

Several structurally-related analogs of JG-98 with further improved selectivity and pharmacokinetic properties have recently been developed, including JG-194, JG-231, JG-294, and JG-345 [20]. Since these compounds all include a rhodacyanine group (Figure S1) that is a known fluorophore, we first tested whether they would be enriched in TT cells using fluorescence microscopy. The results confirmed that the analogs are retained at levels similar to JG-98 and MKT-077 (Figure S2). We next determined IC_50_ values of the analogs in TT and MZ-CRC-1 cells. We found that all of the analogs inhibited growth in TT cell cultures, with JG-194 having the best IC_50_ value (0.92 μM) and JG-231 exhibiting the worst IC_50_ value, which was similar to that of MKT-077 (Figure 1D, left). The effects of these compounds were more varied in MZ-CRC-1 cells. While JG-98, JG-194, and JG-231 exhibited relatively modest potency, the IC_50_ value of JG-194 was about 4-fold lower than that of JG-98 or JG-231 (Figure 1D, right). Although the IC_50_ values of JG-294 and JG-345 were about 2-fold higher than that of JG-98, these values were still 3- to 4-fold lower than the IC_50_ value of MKT-077 in MZ-CRC-1 cells (Figure 1D, right). Together, these data demonstrate that JG-98 analogs can effectively suppress the viability of both MTC cells in vitro.

### 2.2. JG-98 Analogs Effectively Suppress the Viability of Vandetanib- and Cabozantinib-Resistant MTC Cells

**Figure 2:**
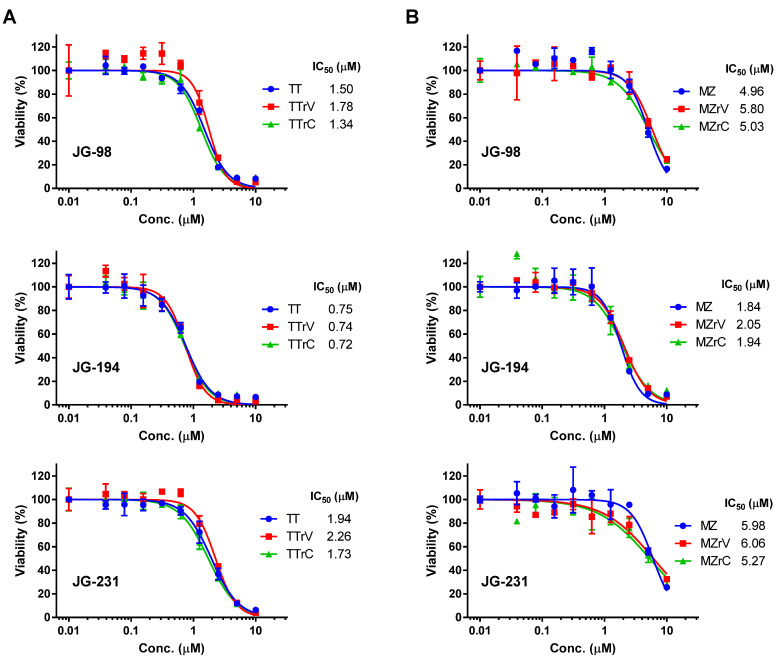
JG-98 analogs suppress the viability of drug-resistant TT and MZ-CRC-1 cells. (**A**) IC_50_ analysis in vandetanib- and cabozantinib-resistant TT cells (TT/rV and TT/rC, respectively) treated with indicated inhibitors for 72 h. (**B**) IC_50_ analysis in vandetanib- and cabozantinib-resistant MZ-CRC-1 cells (MZ/rV and MZ/rC, respectively) treated with indicated inhibitors for 72 h. Data (mean ± SEM, *n* = 3) are expressed as the percentage of vehicle-treated control. Cell viability was determined by MTT assay.

We recently derived vandetanib- and cabozantinib-resistant subpopulations of TT and MZ-CRC-1 cells via prolonged in vitro cultures [14]. As these drug-resistant TT and MZ-CRC-1 cells retained mitochondrial dependency [14], we determined whether JG-98, JG-194, and JG-231 are able to suppress these cells as effectively as their parental cells. Our analyses of the IC_50_ value revealed that vandetanib-resistant TT (TT/rV) and cabozantinib-resistant TT (TT/rC) cells were sensitive to JG-98, JG-194, and JG-231 at similar levels as their parental cells (Figure 2A). Vandetanib-resistant MZ-CRC-1 (MZ/rV) and cabozantinib-resistant MZ-CRC-1 (MZ/rC) cells also exhibited sensitivity to JG-98, JG-194, and JG-231 at similar levels as their parental cells (Figure 2B). Of note, consistent with its effects in parental cells, JG-194 consistently exhibited lower IC_50_ values than JG-98 and JG-231 in these drug-resistant cells. These data demonstrate that the JG-98 analogs can effectively suppress the viability of, not only the drug-naïve, but also the drug-resistant TT and MZ-CRC-1 cells.

### 2.3. Mortalin Depletion Induces Growth Inhibition in TT Cells Grown in Three-Dimensional (3D) Cultures

**Figure 3:**
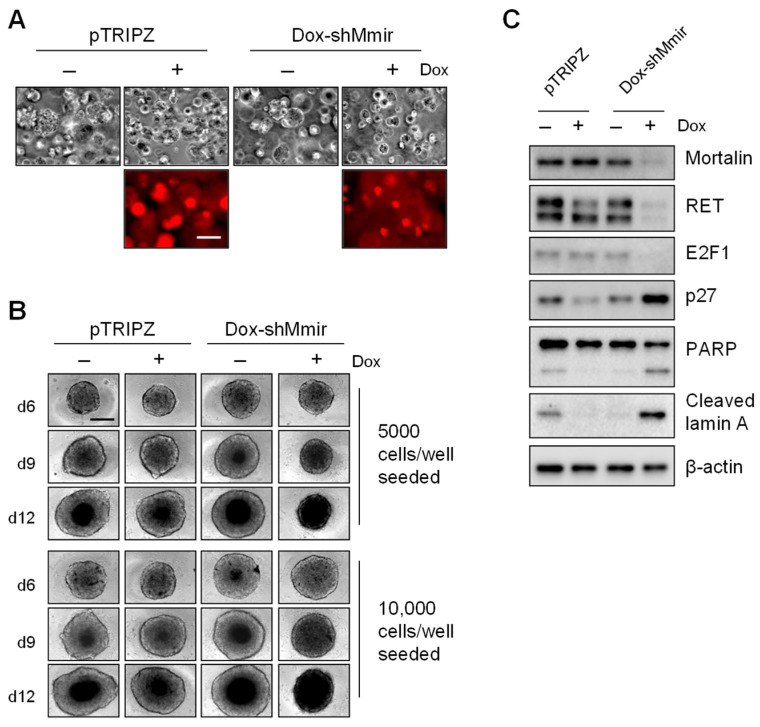
Mortalin knockdown suppresses TT cell growth in three-dimensional cultures. (**A**) Microscopy images of TT cells stably infected with doxycycline (dox)-inducible pTRIPZ virus that expresses shRNA targeting mortalin mRNA (dox-shMmir) or with the control pTRIPZ virus. Cells were treated with 0.5 ug/mL doxycycline (dox) for 9 days in the Matrigel culture. Expression of red fluorescence protein is a visual marking of dox-induced Tet-On activation. Scale bar = 100 µm. (**B**) Microscopy images of TT-dox-shMmir and TT-pTRIPZ cells seeded in 96-well spheroid culture plates at indicated cell density and treated with 0.5 ug/mL doxycycline for 6, 9, and 12 days. Scale bar = 100 µm. (**C**) Western blotting of total lysates of cells described in (**A**). β-actin is the control for equal loading. Microscope images and blots are representative of two independent experiments.

Since 3D cultures better represent in vivo conditions than two-dimensional (2D) cultures and provide more predictive data for in vivo tests [23], we sought to determine whether the JG-98 analogs exert consistent effects on TT and MZ-CRC-1 cells grown in 3D cultures. For this study, we first validated that mortalin depletion suppresses the growth of TT cells in 3D cultures in a manner consistent with our previous observations in 2D cultures [15]. Our data indicated that mortalin knockdown, induced by the lentiviral doxycycline-inducible small hairpin RNA (shRNA) expression system, consistently suppresses the growth of TT spheroid in Matrigel (Figure 3A) and in spheroid culture plates (Figure 3B). Western blot analyses of total lysates of TT cells harvested from the Matrigel culture revealed that mortalin depletion is associated with a marked downregulation of RET and E2F-1; expression of the cyclin-dependent kinase inhibitor, p27^KIP1^; and the cleavage of PARP and lamin A (Figure 3C). These effects of mortalin depletion are identical to previously reported mortalin depletion effects in 2D-cultured TT cells [15], suggesting that these cells respond to mortalin depletion in a consistent manner in 2D- and 3D-culture systems.

### 2.4. JG-98 Analogs Effectively Suppress MTC Cell Growth in 3D Cultures

**Figure 4:**
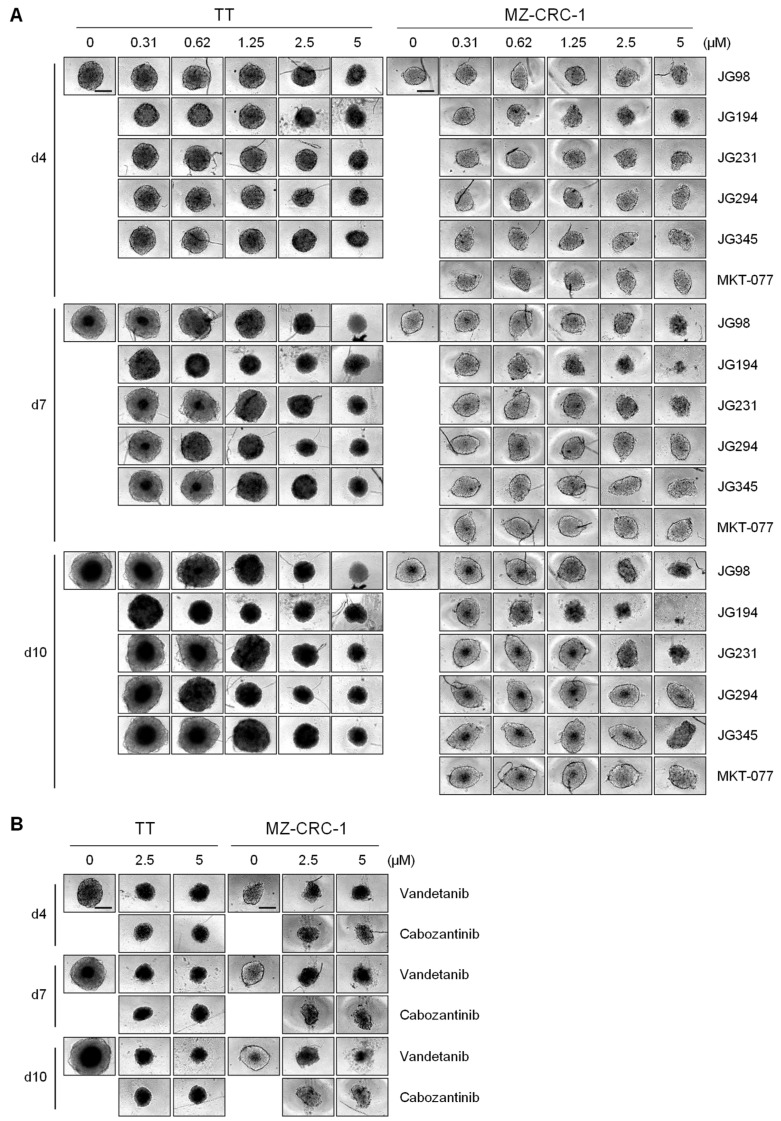
JG-98 analogs suppress TT and MZ-CRC-1 cell growth in spheroid cultures. (**A**) Microscopy images of TT and MZ-CRC-1 cells seeded at 10,000 cells/well in 96-well spheroid culture plates and treated with serially increasing doses of MKT-077 derivatives for 4, 7, and 10 days. (**B**) Microscopy images of TT and MZ-CRC-1 cells seeded at 10,000 cells/well in 96-well spheroid culture plates and treated with vandetanib and cabozantinib for 4, 7, and 10 days. Control cells (0 µM) were treated with equal volume of DMSO. Scale bar = 100 µm. Microscope images are representative of two independent experiments.

We next determined the effects of JG-98 analogs (0.3–5 μM ranges) on TT and MZ-CRC-1 cells grown in spheroid culture plates for 4, 7, and 10 days in comparison with vandetanib and cabozantinib. We found that JG-98 analogs suppressed the growth of TT and MZ-CRC-1 spheroids in a dose- and time-dependent manner (Figure 4A). JG-194 was consistently more effective than other compounds in TT and MZ-CRC-1 3D cultures (Figure 4A), exhibiting comparable potency to equimolar vandetanib and cabozantinib (Figure 4B). Of particular note, JG-194 exhibited even more marked growth inhibitory effects on MZ-CRC-1 spheroids than on TT spheroids (Figure 4A). Although less effective than JG-194, JG-98 and JG-294 showed slightly higher growth inhibitory effects than JG-231 and JG-345 in TT spheroids when observed at later time-points (Figure 4A). In contrast, JG-98 and JG-231 suppressed the growth of MZ-CRC-1 spheroids more effectively than JG-294 and JG-345, while these latter compounds did not show notable growth inhibitory effects in MZ-CRC-1 cells, similarly to MKT-077 (Figure 4A). These effects are consistent with the data obtained from 2D-cultured TT and MZ-CRC-1 cells.

### 2.5. The Effects of JG-231 on TT and MZ-CRC-1 Xenografts in Mice

**Figure 5:**
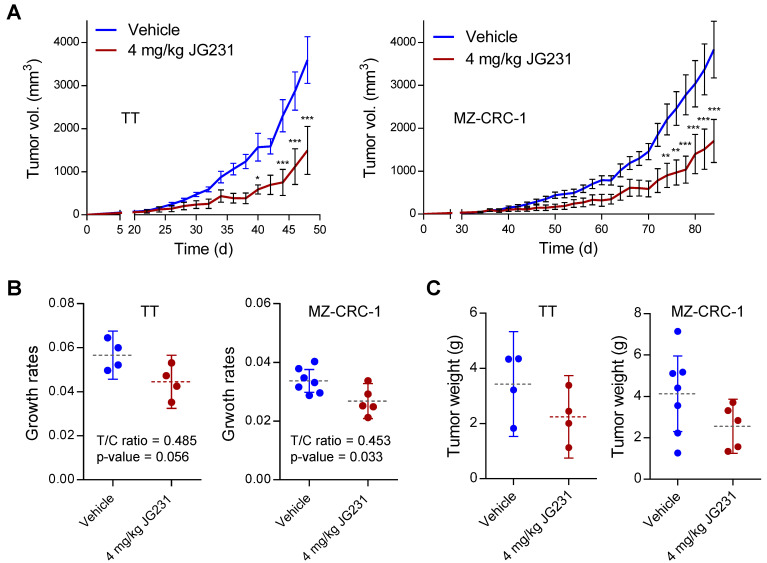

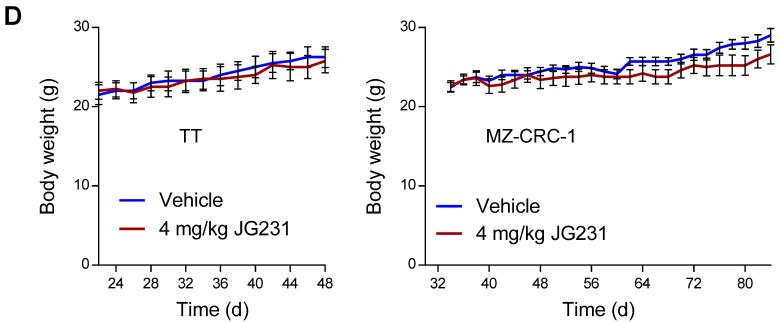
The effects of JG-231 on TT and MZ-CRC-1 xenografts in mice. (**A**) Changes in tumor sizes in athymic mice bearing TT or MZ-CRC-1 xenografts. JG-231 (4 mg/kg/dose) dissolved in 200 μL vehicle was administered intraperitoneally every other day for the indicated treatment period, beginning from day 22 (TT) and day 32 (MZ-CRC-1) after tumor implantation. The control group was treated with the vehicle only. (**B**) Tumor growth rates in (**A**) determined by calculating the rate-based T/C ratios. (**C**) Tumor weights at the time of sacrifice in (**A**). (**D**) Animal body weight changes in (**A**). Data in (**A**,**D**) are mean ± SEM. Horizontal dotted lines and vertical lines in (**B**,**C**) indicate means and 95% Confidence Intervals, respectively. Data are from a single cohort of 4 to 7 mice per treatment group (TT, 4 mice for vehicle, 4 mice for JG-231; MZ-CRC-1, 7 mice for vehicle, 5 mice for JG-231). Each mouse developed one tumor. * *p* < 0.05, ** *p* < 0.01, *** *p* < 0.001 (**A**,**D**, two-way ANOVA with Bonferroni post-tests; (**B**,**C**), two-tailed t-tests).

Observing the improved efficacy of the JG-98 analogs in MTC cells in vitro, we tested whether JG-231 could suppress the growth of TT and MZ-CRC-1 xenografts in athymic nude mice. We chose JG-231 because it has better pharmacokinetic properties than the other JG-98 analogs, such as JG-194 [20]. When administered intraperitoneally at 4 mg/kg dose every other day, JG-231 delayed the growth of TT and MZ-CRC-1 xenografts in mice (Figure 5A). Moreover, tumor growth rates calculated by the rate-based T/C ratios method [24] suggested that JG-231 could significantly decrease tumor growth rates in MZ-CRC-1 tumors (Figure 5B). Similar trends were seen in TT xenografts, but statistical significance could not be achieved (*p*-value 0.056), likely due to the limited sample size. In both models, tumor weights measured at the end of the studies were slightly decreased in the treated groups, although the data were not significant due to wide variation (Figure 5C). Of note, compared to our previous observation with MKT-077 [22], treatment with JG-231 did not lead to loss of body weight (Figure 5D). This improved safety is likely a product of the increased potency and improved pharmacokinetics of JG-231, which allows a 2-fold decrease in dosing. Together, these in vivo data are consistent with the in vitro findings described above, supporting the anti-proliferative effects of the JG-98 analogs in MTC cells.

## 3. Discussion

We previously demonstrated that mortalin is an essential factor in the maintenance of mitochondrial bioenergetics in MTC cells [15]. We also showed that treatment with MKT-077 suppresses TT xenografts in immune-compromised mice, when administered at a 8 mg/kg dose with a day interval [22]. Although this result identified MKT-077 as a promising lead, its potential was limited because MKT-077 treatment resulted in significant weight loss and general toxicity in animals, which is consistent with its previously reported toxicity in human patients [18]. Moreover, MKT-077 was not effective in MZ-CRC-1 cells [22]. The present study demonstrates that the newer benzothiazole rhodacyanine derivatives of MKT-077, such as JG-98, JG-194, and JG-231, can effectively suppress TT and MZ-CRC-1 cells in vitro and in vivo.

Previous in vitro analyses showed that MKT-077 and its analogs interact with an allosteric binding pocket that is conserved amongst the major HSP70 paralogs [17,25,26]. As mortalin is mainly located in mitochondria in MTC cells [15], we speculate that mitochondrial partitioning is a key criterion for the potency of the MKT-077 derivatives in MTC cells, although other factors, including import/export kinetics, ‘off-target’ effects, and other as-yet-unidentified mechanisms may also be involved. In support of an important role for mitochondrial localization, our current data show that YM-08 is not as effective as MKT-077 in suppressing the viability of MTC cells. Although both compounds were shown to occupy the same binding sites on HSP70 [19], only MKT-077 and the other analogs partition to the mitochondria via their cationic pyridinium. Moreover, we recently demonstrated that mortalin overexpression confers resistance to JG-98 and JG-231 in cells, while mortalin depletion sensitizes cells to these compounds, strongly supporting the selectivity of these compounds [27]. Together, these results support the important, but perhaps not exclusive, role of mortalin inhibition in the anti-proliferative activity of compounds in this chemical series.

More broadly, we previously found that TT cells are more sensitive to mitochondrial membrane potential (Δψ_m_)-sensitive agents, such as triphenyl-phosphonium-carboxy-proxyl and mitoquinone than MZ-CRC-1 cells [13,14]. Consistent with this idea, our data show that MZ-CRC-1 cells are generally less sensitive to the MKT-077 derivatives, which are also driven by Δψ_m_ [16,17], than TT cells. Thus, evaluation of mitochondrial activity may predict MTC cell responsiveness to a broader set of therapeutic agents that target mitochondria.

YM-08 can interact with HSP70 proteins, even with higher affinity in vitro, and suppress proliferation of a few breast cancer cell lines, albeit somewhat less effectively than MKT-077 [19]. Of note, the 40-fold difference in IC_50_ values between MKT-077 and YM-08 in TT cells is in marked contrast to the previously reported differences in IC_50_ values of these compounds in MCF7 (<5-fold) and MDA-MB-231 (<7-fold) breast cancer cell lines [19]. This suggests that the relative potency of analogs in this series can vary in different cell types. In typical medicinal chemistry campaigns, the goal is to identify a single compound that is the most potent and metabolically stable. Such a ‘lead’ compound is then deployed in other disease models. However, because of differences in mitochondrial potential, expression of drug efflux proteins and other factors, it seems possible (or even likely) that the absolute rank-ordering of compounds in a series might be different in different cell lines. In our case, the MKT-077 analogs were designed for potency in breast and prostate cancer cell lines [20]. However, we hypothesized that their relative potency might be partially distinct in MTC models. While the mechanisms of these differences would be hard to fully elucidate, it may be possible that these compounds are differentially partitioned into subcellular locations and/or affect distinct HSP70 paralogs, to varied degrees. This activity against other HSP70 paralogs could be particularly interesting. For example, it has been shown that the ER-resident paralog, BiP, is also upregulated in MTC [28], although its significance has not yet been determined. In screens of a subset of JG-98 analogs (see Figure S1), we found that JG-194 was the most potent, unlike in other cancers [20]. One broader lesson from these studies might be to test multiple, structurally similar analogs in each cell model, to understand whether the structure–activity relationships (SARs) are distinct.

The anti-proliferative effects of JG-194 in MTC cells were quite comparable to the effects of vandetanib and cabozantinib used at equivalent dosages. Moreover, JG-194 was also effective in the vandetanib- and cabozantinib-resistant progenies of these cells. Together, those findings suggest that mortalin is a promising target for further development. However, achieving this goal will likely require additional synthetic efforts. For example, although 4 mg/kg JG-231 effectively suppressed both TT and MZ-CRC-1 xenografts in mice, with modest effects on bodyweight loss (compared to MKT-077 in TT xenografts [22]), it was less effective than JG-194 in vitro. Unfortunately, JG-194 has poor metabolic stability, so further studies into this scaffold, such as recent work [29], in the context of MTC inhibition is warranted.

In conclusion, our current data demonstrate an advance in mortalin targeting in MTC cells and, consistent with our previous observations, support the potential of mortalin as a target for the design of a molecular therapy for MTC.

## 4. Materials and Methods

### 4.1. Cell Culture and Reagents

The human MTC lines, TT and MZ-CRC-1, were maintained as previously described [30,31]. Briefly, TT was maintained in RPMI 1640 (Gibco, Waltham, MA, USA, 11835-030) supplemented with 16% fetal bovine serum (Gibco, Waltham, MA, USA, SH30541.03), 100 U/mL penicillin, and 100 μg/mL streptomycin (Gibco, Waltham, MA, USA, 15140-122). MZ-CRC-1 was maintained in high-glucose DMEM (Gibco, Waltham, MA, USA, 11965-118) supplemented with 10% FBS in culture dishes coated with rat collagen (Sigma, St. Louis, MO, USA, 122-20). Generation of TT-dox-shMmir, TT cells stably infected with doxycycline (dox)-inducible pTRIPZ virus that expresses shRNA targeting mortalin mRNA (dox-shMmir), was previously described [15]. For spheroid formation, cells were plated at 5000 or 10,000 cells/well in the RPMI 1640 medium supplemented as above onto 96-well Corning Spheroid Microplates (Corning, Tewksbury, MA, USA, #4515), and medium was changed every three days. For cell culture in Matrigel matrix, 10,000 cells were plated onto 24-well plates precoated with 200 µL Matrigel (Corning, Tewksbury, MA, USA, #356231) and maintained with the RPMI 1640 medium mixed with Matrigel at 10% of final volume (0.8–1.1 mg/mL), as instructed by the manufacturer. All experiments were performed using cells within 10 passages from the point of acquisition. Vandetanib and cabozantinib were purchased from LC Laboratories (Woburn, MA, USA, V-9402) and Selleckchem (Houston, TX, USA, BMS-907351), respectively. Compounds, including MKT-077, JG-98, and their analogs, were synthesized and characterized for identity by ^1^H NMR and LC-MS/MS and purity by HPLC (>95%), as previously described [20].

### 4.2. Analysis of Cell Viability

Cell viability was measured by performing a colorimetric 3-(4,5-dimethyl-2-thiazolyl)-2,5-diphenyltetrazolium bromide (MTT, Sigma, St. Louis, MO, USA) assay, as previously described [32]. Briefly, cells were seeded in 24-well plates and allowed to attach for 48 h. After drug treatment, cells were incubated with 400 μL of MTT (0.5 mg/mL) in complete medium for 2 h at 37 °C, switched into 200 μL of dimethyl-sulfoxide, and shaken for 5 min at room temperature, before measuring absorbance at 540 nm.

### 4.3. Immunoblotting

Cells were harvested in lysis buffer containing 62.5 mM Tris-HCl (pH 6.8)/2% SDS, and protease and phosphatase inhibitor cocktails 2 and 3 (Sigma-Aldrich, St. Louis, MO, USA, P8340, P5726, P0044). Protein concentrations were measured using the bicinchoninic acid reagent (Pierce, Waltham, MA, USA, 23228, 1859078). Proteins were resolved by sodium dodecyl sulfate polyacrylamide gel electrophoresis and transferred to polyvinylidene difluoride membrane filter (Bio-Rad, Hercules, CA, USA, 1620177). After transfer, membranes were blocked at 25 °C for 1 h in buffer containing 0.1 M Tris (pH 7.4), 0.9% NaCl, 0.05% Tween 20, and 5% nonfat dry milk.

Membranes were then incubated with the appropriate antibodies overnight at 4 °C, at the dilutions indicated as follows: poly(ADP-ribose) polymerase (PARP, #9542), 1:1000; cleaved lamin A (#2035), 1:2000; cytochrome c oxidase subunit IV (COX IV, #4850), 1:2000 (Cell Signaling, Danvers, MA, USA); β-actin A2228) 1:5000 (Sigma, St. Louis MO, USA, A1978); E2F-1 (sc-193), 1:1000; RET (sc-167), 1:1000; mortalin (sc-133137), 1:5000; p27^KIP1^ (sc-1641) 1:1000 (Santa Cruz Biotechnology, Santa Cruz, CA, USA). SuperSignal West Pico and Femto chemiluminescence kits (Pierce, Waltham, MA, USA, 34579 and 34094) were used for visualization of the signal. For densitometry, immunoblots were analyzed using Image Lab software (Bio-Rad, Hercules, CA, USA).

### 4.4. Tumor Xenograft Studies

A total of 1 × 10^7^ TT cells in 200 µL HBSS were inoculated subcutaneously into the rear flanks of 6-week-old female athymic nude (*nu/nu*) mice (Charles River Laboratories). Once palpable, tumors were measured using Vernier calipers at the intervals indicated in the text. Tumor volumes (TV) were calculated using the formula: TV = L × W^2^ × 0.5 (L = length, W = width). When TV reached 100 mm^3^, mice were sorted into groups of 8 to achieve equal distribution of tumor size in all treatment groups. Group 1 received only the vehicle (1:9 mixture of DMSO/saline) and group 2 received JG-231 (4 mg/kg body weight/dose). Then, 200 μL of ether solution was administered by intraperitoneal (*i.p.*) injection every two days (total 10 doses). At the end of the experiments, animals were euthanized by CO_2_ asphyxiation. All animal studies were performed according to protocols approved by the Institutional Animal Care and Use Committee at Medical College of Wisconsin (AUA00001327). Tumor growth rates were determined by calculating the rate-based T/C ratios, as previously described [24].

### 4.5. Statistical Analysis

Statistical significance was determined by two-way ANOVA with Bonferroni post-tests and two-tailed unpaired Student’s *t*-test using PRISM (Graph-Pad Software, La Jolla, CA, USA). IC_50_ and Confidence Intervals were determined by PRISM (Graph-Pad Software, La Jolla, CA, USA). *p* values of <0.05 were considered statistically significant.

## Data Availability

Not applicable.

## Supplementary Materials

The following supporting information can be downloaded at: https://www.mdpi.com/article/10.3390/ijms23031063/s1.
