# Supplementary material for: Analogs of the Heat Shock Protein 70 Inhibitor MKT-077 Suppress Medullary Thyroid Carcinoma Cells
Source: Int J Mol Sci. 2022 Jan 19;23(3):1063. doi: 10.3390/ijms23031063 (PMC8835675; doi:10.3390/ijms23031063)
Supplement: Supplementary file 1 [file ijms-23-01063-s001.zip › ijms-1541668-supplementary.pdf]

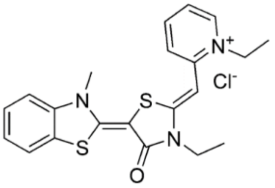

MKT-077

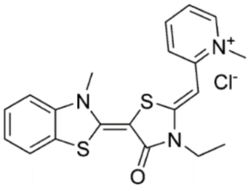

YM-01

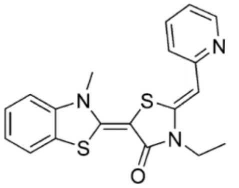

YM-08

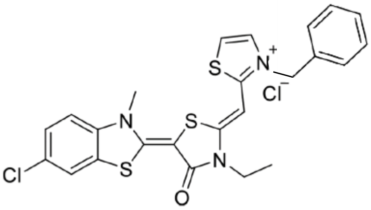

JG98

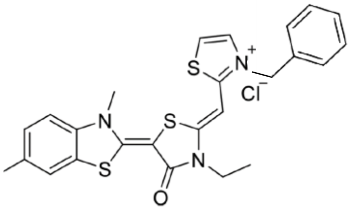

JG194

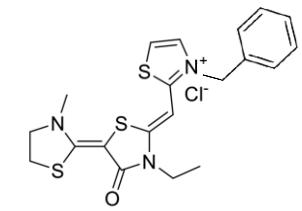

JG258

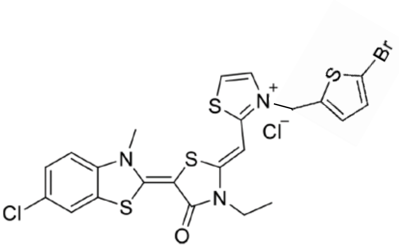

JG231

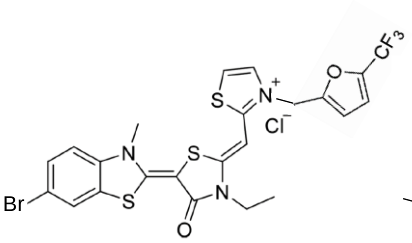

JG294

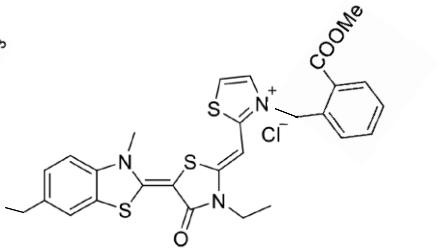

JG345

Figure S1. Chemical structures of MKT-077 derivatives used in this study

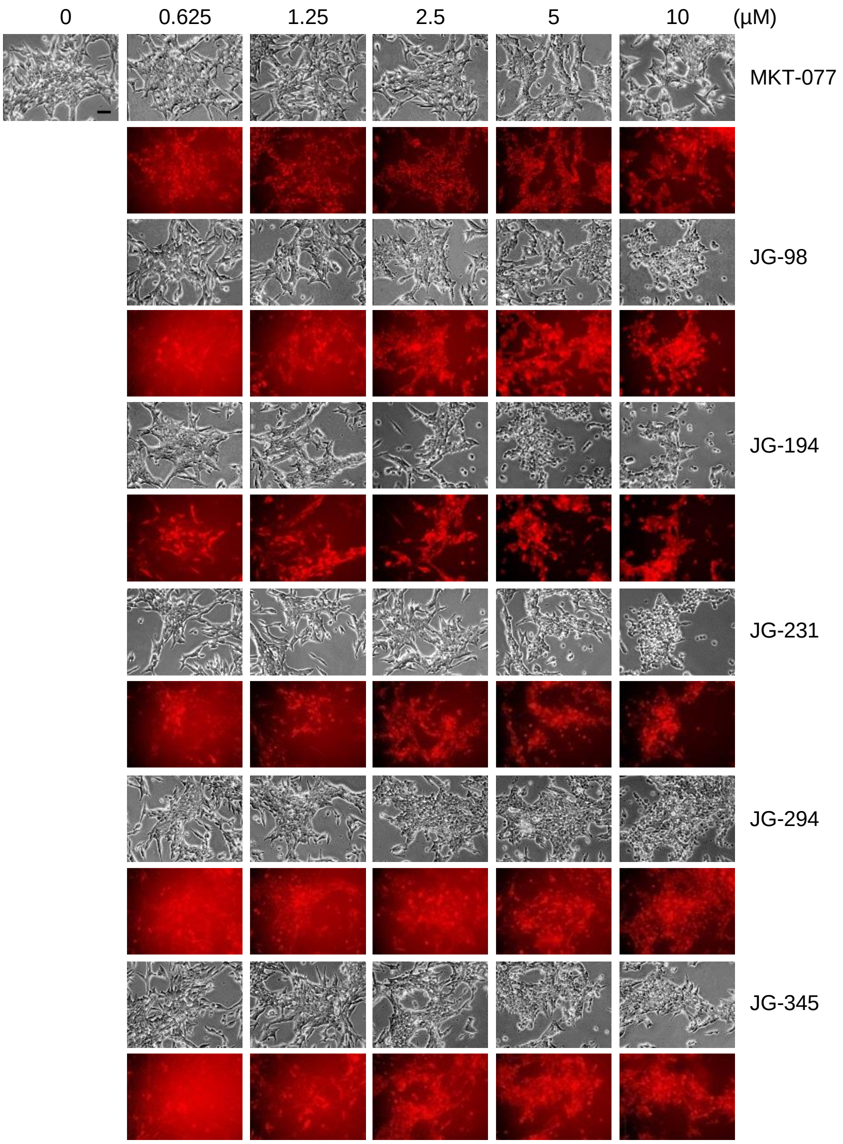

Figure S2. Images under a fluorescent microscope (scale bar = 100 µm) of TT cells treated with the indicated chemical compounds for 24 hour.
